# Spent Hen Muscle Protein-Derived RAS Regulating Peptides Show Antioxidant Activity in Vascular Cells

**DOI:** 10.3390/antiox10020290

**Published:** 2021-02-15

**Authors:** Hongbing Fan, Khushwant S. Bhullar, Jianping Wu

**Affiliations:** 1 Department of Agricultural, Food and Nutritional Science, 4-10 Ag/For Building, University of Alberta, Edmonton, AB T6G 2P5, Canada; hongbing@ualberta.ca (H.F.); bhullar@ualberta.ca (K.S.B.); 2 Cardiovascular Research Centre, University of Alberta, Edmonton, AB T6G 2R7, Canada; 3 Department of Pharmacology, 7-55 Medical Sciences Building, University of Alberta, Edmonton, AB T6G 2H7, Canada

**Keywords:** chicken, laying hen, muscle protein, ACE, ACE2, oxidative stress, antioxidant peptides, antioxidant enzymes

## Abstract

Spent hens are egg-laying hens reaching the end of their egg-laying cycles, being a major byproduct of the egg industry. Recent studies have been focusing on finding new value-added uses for spent hens. We have previously identified four bioactive peptides from spent hen muscle proteins, including three angiotensin-converting enzyme (ACE) inhibitory (ACEi) peptides (VRP, LKY, and VRY), and one ACE2 upregulating (ACE2u) peptide (VVHPKESF (V-F)). In the current study, we further assessed their antioxidant and cytoprotective activities in two vascular cell lines—vascular smooth muscle A7r5 cells (VSMCs) and endothelial EA.hy926 cells (ECs)—upon stimulation by tumor necrosis factor alpha (TNFα) and angiotensin (Ang) II, respectively. The results from our study revealed that all four peptides attenuated oxidative stress in both cells. None of the investigated peptides altered the expression of TNFα receptors in ECs; however, VRY and V-F downregulated Ang II type 1 receptor (AT_1_R), while V-F upregulated the Mas receptor (MasR) in VSMCs. Further, we found that the antioxidant effects of VRP, LKY, and VRY were likely through acting as direct radical scavengers, while that of V-F was at least partially ascribed to increased endogenous antioxidant enzymes (GPx4 and SOD2) in both cells. Besides, as an ACE2u peptide, V-F exerted antioxidant effect in a MasR-dependent manner, indicating a possible involvement of the upregulated ACE2-MasR axis underlying its antioxidant action. The antioxidant activities of VRP, LKY, VRY, and V-F in vascular cells indicated their multifunctional properties, in addition to their ACEi or ACE2u activity, which supports their potential use as functional food ingredients against hypertension.

## 1. Introduction

Hypertension is the leading cause of global morbidity and mortality, afflicting more than 20% of adults [1,2]. Currently, pharmaceutical drugs are the major treatment for hypertension, but prolonged use of pharmacological treatment is expensive and is generally associated with various adverse effects, such as dry cough and angioedema [3,4]. In the wake of economic and health-related distresses, lifestyle changes, such as increasing physical activity and adopting suitable dietary approaches, to stop hypertension are being recommended [1]. Food-derived bioactive compounds, such as antihypertensive peptides, have been an emerging treatment for the prevention and treatment of hypertension [5].

Despite the complicated pathogenesis of hypertension, the hyperactive renin–angiotensin system (RAS), endothelial dysfunction, and aberrant inflammation accompanied by excessive oxidative stress have been identified as important pathological contributors [6,7,8,9]. In the vasculature, redox stress and resultant oxidative damage are mediators of vascular impairment and inflammation in many cardiovascular diseases, including hypertension [10]. Anatomically, vascular smooth muscle cells (VSMCs) and endothelial cells (ECs) are two major components of the vascular wall, both with the ability to produce reactive oxygen species (ROS) upon stimulation by various cytokines, such as angiotensin (Ang) II and tumor necrosis factor alpha (TNFα) [10]. For example, Ang II, a key active profibrogenic cytokine in the RAS, not only induces vasoconstriction and inflammation, but also stimulates ROS production via Ang II type 1 receptor (AT_1_R) binding [11]. ROS trigger intracellular signaling, such as the nuclear factor kappa B (NF-κB) pathway, a known mediator of inflammation, and react with nitric oxide, a key vasodilator, resulting in systematic vascular dysfunction [12,13,14,15]. Genetic models of hypertension, such as spontaneously hypertensive rats (SHRs), are reported to have increased ROS production in their vasculature, while supplementation with antioxidant agents, such as α-tocopherol and ascorbic acid, can attenuate oxidative stress, improve vascular function, and prevent the development of hypertension [16,17,18]. Likewise, food-derived antioxidant peptides have shown abilities in ameliorating excessive oxidative stress in various cell and animal models of hypertension [19,20,21,22,23]. Several spent hen-derived peptides, including IWHH, IWH, and IW, have been reported to reduce the basal oxidative stress in ECs [24].

Spent hens are laying hens that reach the end of the egg-laying cycle and are a major byproduct of the egg industry. In 2019, approximately 400 million spent hens required disposal in North America [25,26]. Despite being a byproduct, spent hen contains various muscle proteins that can be used to produce bioactive peptides with appropriate processing and value addition. We previously prepared a spent hen muscle protein hydrolysate (using a food-grade protease: thermoase PC10F) that exhibited antihypertensive activity in SHR [27]. The hydrolysate showed great potential in modulating the RAS, with potent angiotensin-converting enzyme (ACE) inhibitor (ACEi) activity in vitro, and ACE2 upregulating (ACE2u) activity in VSMCs; also, four peptides, from a major muscle protein, myosin, have thus been identified, including three ACEi peptides (VRP, LKY, and VRY) and one ACE2u peptide (VVHPKESF (V-F)) [28]. Prior to evaluating their antihypertensive effects in SHR, we aimed to first study their antioxidant activities, since peptides are reported to reduce blood pressure through multiple mechanisms including the amelioration of vascular oxidative stress [29]. In addition, antihypertensive agents with a single pharmacological effect have been reported to be inadequate for blood pressure control, which usually requires combined use with other pharmaceutical agents or bioactives [4]. Given the critical implications of vascular oxidative stress in hypertension, two vascular cell lines, VSMCs (A7r5) and ECs (EA.hy926), were selected for this study [9,10,30]. The current study aims to explore the antioxidant effects of VRP, LKY, VRY, and V-F in VSMCs and ECs, as well as the underlying mechanisms underlying their antioxidant actions.

## 2. Materials and Methods

### 2.1. Materials

Peptides, including VRP, LKY, VRY, and V-F (purity > 98%) were synthesized by Genscript Corp (Piscataway, NJ, USA). Triton-X-100, dithiothreitol (DTT), and Ang II were obtained from Sigma (St Louis, MO, USA). Losartan potassium and dihydroethidium (DHE) were purchased from Tocris (Oakville, ON, Canada) and Biotium (Fremont, CA, USA), respectively. TNFα and A779 were obtained from R&D Systems (Minneapolis, MN, USA). Nonessential amino acids (NEAAs), penicillin−streptomycin, 4-(2−68 hydroxyethyl)-1-piperazineethanesulfonic acid (HEPES), 0.25% (*w*/*v*) trypsin-0.53 mM ethylenediaminetetraacetic acid (EDTA), Dulbecco’s modified Eagle’s medium (DMEM), and fetal bovine serum (FBS) were purchased from Gibco Invitrogen (Burlington, ON, Canada). ECs (EA.hy926, CRL-2922) and VSMCs (A7r5, CRL-1444) were provided by the American Type Culture Collection (Manassas, VA, USA).

### 2.2. Cytotoxicity of VRP, LKY, VRY, and V-F in Vascular Cells

The cytotoxicity of peptides against VSMCs and ECs followed an alamarBlue assay, as depicted in Fan et al. [31]. After reaching 80% of confluency on a 96-well plate, cells were treated with 100 μM of peptides for 24 h in growth media, after which the media were replaced with 200 μL of 10% alamarBlue solution (in growth medium, *v*/*v*) for 4 h avoiding light. Then, 150 μL of the solution was transferred into an opaque 96-well plate for fluorescence signal detection, with an emission wavelength at 590 nm and excitation wavelength at 560 nm. A control without any treatment was added.

### 2.3. Cell Culture of VSMCs and ECs

Both cells were grown in DMEM containing 10% FBS and 1% penicillin–streptomycin at 37 °C in with 5% CO_2_ and 100% humidity; ECs were supplemented with NEAAs (1%). The growth media were changed every three days for both cells.

### 2.4. Superoxide Detection

Superoxide detection was detected in VSMCs and ECs by DHE staining, as described in Fan et al. [27] with slight modifications. VSMCs were treated with peptides (50 µM) for 1 h or 24 h before adding 1 μM of Ang II for 30 min. An AT_1_R antagonist (losartan potassium, 50 µM) or Mas receptor (MasR) antagonist (A779, 1 µM) might be added 1 h before Ang II treatment [32]. ECs were treated with peptides (50 µM) for 18 h before adding 10 ng/mL of TNFα for 30 min. Then, DHE (20 μM) was added for 30 min (protected from light). Cells were then washed three times with a non-phenol-red DMEM before detecting the fluorescence signal under an Olympus IX81 fluorescent microscope (Olympus, Tokyo, Japan). Each data point was comprised of two or three random fields, whose total fluorescence intensity was obtained using ImageJ software (https://imagej.net/Welcome, accessed on 2 January 2021) while the mean fluorescence intensity per cell (MFI/cell) was calculated. The control (untreated group) had no treatment.

### 2.5. Lipid Peroxidation Assay

The determination of malondialdehyde (MDA) in cells was performed according to the manufacturer’s instructions of the lipid peroxidation assay kit provided by Abcam (ab118970). Briefly, both VSMCs and ECs were seeded at a concentration of 2 × 10^6^ cells in a 96-well plate and allowed to reach ~70% confluency before treatment. VSMCs were treated with peptides (50 µM) for 24 h, and ECs were treated with peptides (50 µM) for 18 h, followed by oxidative stress using 1 μM of Ang II or 10 ng/mL of TNFα for 30 min, respectively. After the treatment, both cells were washed twice with cold PBS, and homogenized in 300 µL lysis solution per well (Buffer + butylated hydroxytoluene (BHT)). Samples were centrifuged at 13,000× *g* for 10 min to collect the supernatant. The 200 µL of supernatant was transferred to a fresh tube, and 600 µL of thiobarbituric acid (TBA) reagent was added. This mixture-containing tube was incubated at 95 °C for 60 min, and then immediately cooled to room temperature in an ice bath for 10 min. After cooling, 200 µL of supernatant was added to a 96-well plate, and the absorbance was detected immediately on a microplate reader at 532 nm. The final results were presented as malondialdehyde (nmol/mL).

### 2.6. Western Blotting

Cells were placed in quiescing media (containing 1% FBS) until reaching confluency. VSMCs were treated with peptides (50 µM) for 24 h for the detection of AT_1_R and MasR; ECs were treated with peptides (50 µM) for 18 h for the detection of TNFα receptors 1/2 (TNF-R1/R2). Peptide concentrations and time of treatment were selected per our previous studies [28,33]. After the treatment, cells were scraped and lysed in a boiling Laemmle’s buffer with 50 mM DTT and 0.2% Triton-X-100.

Cell samples were run in a 9% separating gel and transferred to a nitrocellulose membrane before being incubated with specific primary antibodies. Protein bands of AT_1_R (PA5-20812, Invitrogen), MasR (NBP1-78444, Novus Biologicals, Toronto, ON, Canada), TNF-R1 (sc-8436, Santa Cruz, Dallas, TX, USA), TNF-R2 (sc-8041, Santa Cruz), glutathione peroxidase 4 (GPx4; ab125066, Abcam, Toronto, ON, Canada), and superoxide dismutase 2 (SOD2; ab227091, Abcam) were normalized to α-tubulin (ab15246, Abcam) or glyceraldehyde 3-phosphate dehydrogenase (GAPDH, ab8245, Abcam). The fluorescent bands were visualized by adding donkey-anti-mouse IRDye 680 RD or donkey-anti-rabbit 800 CW secondary antibodies (Licor Biosciences, Lincoln, NE, USA), and the signals were detected using Licor Odyssey BioImager (Licor Biosciences).

### 2.7. Statistical Analysis

Data were expressed as mean ± standard error of the mean (SEM) of three to four independent experiments, unless otherwise mentioned, and were subjected to one-way analysis of variance (ANOVA) with Dunnett’s multiple test, using GraphPad version 6 (San Diego, CA, USA). A value of *p* < 0.05 was considered to be statistically different.

## 3. Results

### 3.1. Cytotoxicity of VRP, LKY, VRY, and V-F in ECs and VSMCs

**Figure 1:**
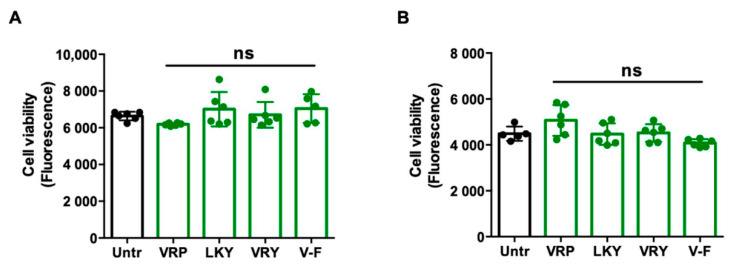
Cytotoxicity of VRP, LKY, VRY, and VVHPKESF (V-F) in ECs (**A**) and VSMCs (**B**). Both cells were treated with 100 μM of peptides for 24 h, followed by an alamarBlue cell viability assay. Data were expressed as means ± standard error of the mean (SEM) of 5–6 independent experiments; ns: not significant (*p* > 0.05), as compared to the untreated group (Untr).

Prior to evaluating the antioxidant activity of the four peptides in cells, their effects on cell viability were first determined. Incubation of the peptides for 24 h at 100 µM, higher than the tested concentration of peptides (50 µM) used in the subsequent experiments, indicated no effect on cell viability for either ECs and VSMCs in vitro (Figure 1A,B).

### 3.2. Antioxidant Effect of VRP, LKY, VRY, and V-F in ECs and VSMCs

**Figure 2:**
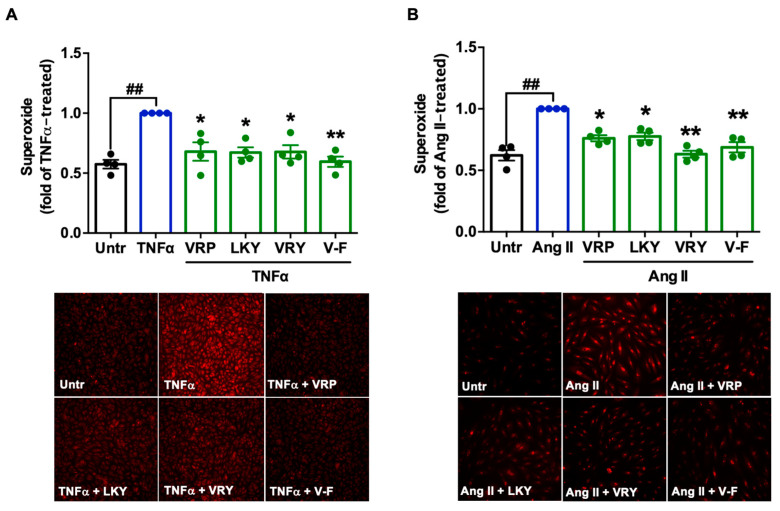
Effect of VRP, LKY, VRY, and V-F on superoxide generation in ECs (**A**) and VSMCs (**B**). Cells were treated with peptides (50 μM) for 18 h before treatment with 10 ng/mL of TNFα for 30 min (or for 1 h before treatment with 1 μM of angiotensin (Ang) II for 30 min). Data were expressed as means ± SEMs of four independent experiments, normalized to the tumor necrosis factor (TNF)α-/Ang II-treated group. ## *p* < 0.01, * *p* < 0.05, ** *p* < 0.01, compared to the TNFα-/Ang II-treated group. Untr, untreated group.

**Figure 3:**
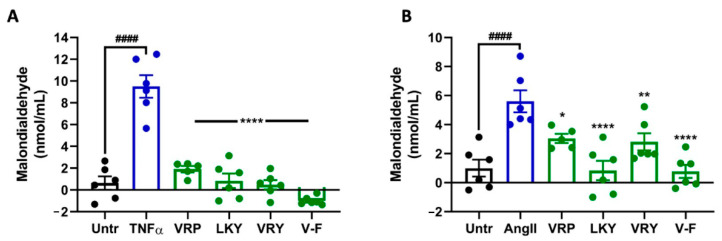
Effect of VRP, LKY, VRY, and V-F on formation of malondialdehyde (MDA) in ECs (**A**) and VSMCs (**B**). Cells were treated with peptides (50 μM) for 18 h before treatment with 10 ng/mL of TNFα for 30 min (or for 24 h before treatment with 1 μM of Ang II for 30 min). Data were expressed as means ± SEM of 5–6 independent experiments, normalized to the TNFα-/Ang II-treated group. #### *p* < 0.0001, * *p* < 0.05, ** *p* < 0.01, and **** *p* < 0.0001, as compared to the TNFα-/Ang II-treated group. Untr, untreated group.

Figure 2 shows the cytoprotective effect of four peptides on superoxide production in TNFα-stimulated ECs or Ang II-stimulated VSMCs in vitro. TNFα or Ang II treatment significantly generated superoxide in both vascular cells (*p* < 0.01). All four peptides were able to diminish superoxide generation in both cells (*p* < 0.05). MDA is one of the major end products formed due to lipid peroxidation triggered by oxidative stress; the results showed that the four peptides significantly reduced MDA levels in both cells, further indicating the antioxidant effect of these peptides (Figure 3A,B). The ability of LKY and V-F to reduce superoxide following Ang II stress in VSMCs was significantly higher than VRP and VRY (Figure 3B).

### 3.3. Effect of VRP, LKY, VRY, and V-F on the Expression of TNF-R1/R2 in ECs

**Figure 4:**
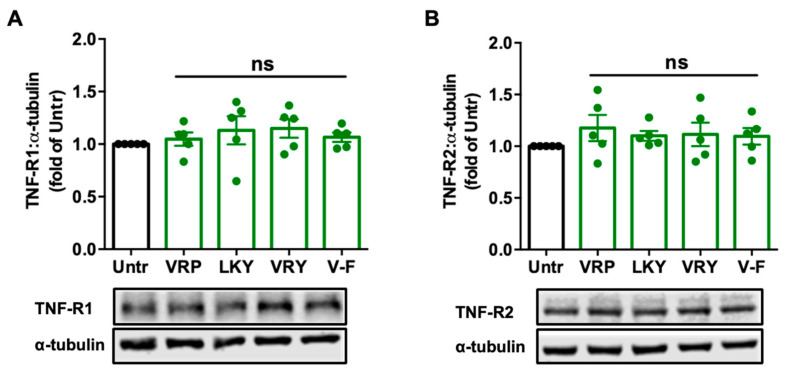
Effect of VRP, LKY, VRY, and V-F on the expression of TNF-R1 (**A**) and TNF-R2 (**B**) in ECs. Cells were treated with 50 μM of peptides for 18 h. Protein bands were quantified by densitometry and normalized to α-tubulin. Data were expressed as means ± SEM of five independent experiments and normalized to the untreated group (Untr); ns: not significant (*p* > 0.05), as compared to the untreated group.

Since TNFα induces ROS production and oxidative stress, mainly via binding with TNF receptors, we further studied the modulatory effect of peptides on TNF-R1 and TNF-R2 in ECs. Results showed that treatments with these peptides for 18 h did not alter the expression of the two receptors in TNFα-stimulated ECs in vitro (Figure 4); similar results were observed by further increasing the treatment period to 48 h (Figure S1).

### 3.4. Effects of VRP, LKY, VRY, and V-F on the Expression of AT_1_R and MasR Receptors in VSMCs

**Figure 5:**
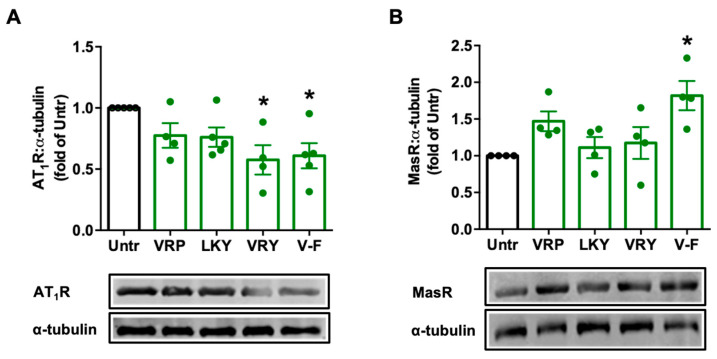
Effect of VRP, LKY, VRY, and V-F on the expressions of AT_1_R (**A**) and MasR (**B**) in VSMCs. Cells were treated with peptides (50 μM) for 24 h. Protein bands were quantified by densitometry and normalized to α-tubulin. Data were expressed as means ± SEM of 4–5 independent experiments, normalized to the untreated group (Untr); ns: not significant (*p* > 0.05); * *p* < 0.05, as compared to the untreated group.

Ang II stimulates ROS production via AT_1_R in VSMCs; however, Ang II can be degraded by ACE2 to form Ang (1–7), and the latter, via MasR, attenuates the harmful effect from the Ang II–AT_1_R axis, including oxidative stress. We found that VRY and V-F significantly downregulated AT_1_R expression (*p* > 0.05) (Figure 5A), while only V-F significantly upregulated MasR expression (*p* < 0.05) (Figure 5B).

### 3.5. Involvement of AT_1_R or MasR in Antioxidant Effect of VRP, LKY, VRY, and V-F in VSMCs

**Figure 6:**
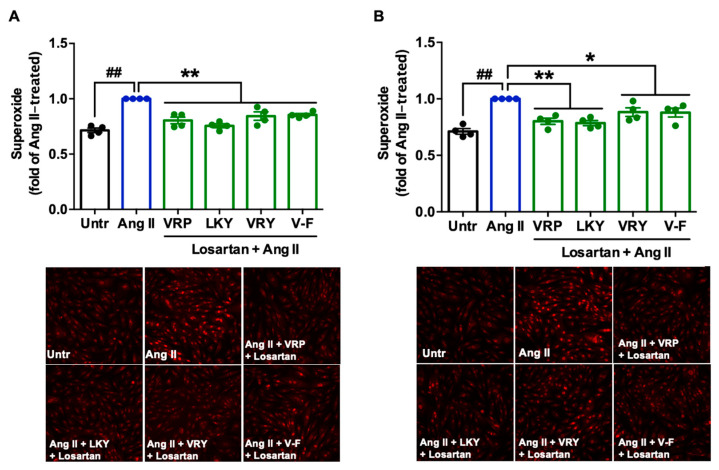
Involvement of AT_1_R in the antioxidant effect of VRP, LKY, VRY, and V-F in Ang II-stimulated VSMCs. Peptides (50 μM) were incubated with cells for (**A**) 1 h, with 50 μM AT_1_R antagonist (losartan), or (**B**) 24 h, followed by adding 50 μM losartan for 1 h, before the addition of Ang II (1 μM) for 30 min. Data were expressed as means ± SEM of four independent experiments and normalized to the Ang II-treated group. ## *p* < 0.01, * *p* < 0.05, ** *p* < 0.01, as compared to the Ang II-treated group. Untr, untreated group.

**Figure 7:**
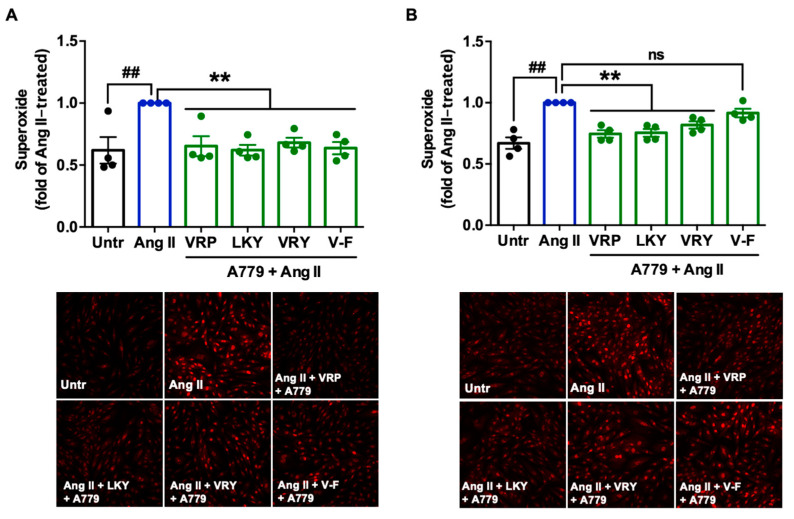
Involvement of MasR in the antioxidant effect of VRP, LKY, VRY, and V-F in Ang II-stimulated VSMCs. Peptides (50 μM) were incubated with cells for (**A**) 1 h with 1 μM MasR antagonist (A779) or (**B**) 24 h followed by adding 1 μM A779 for 1 h, before the addition of Ang II (1 μM) for 30 min. Data were expressed as means ± SEMs of 4 independent experiments and normalized to the Ang II-treated group. ##, *p* < 0.01, **, *p* < 0.01, ns, not significant (*p* > 0.05), as compared to the Ang II-treated group. Untr, untreated group.

Since two peptides (VRY and V-F) regulated the expression of AT_1_R or MasR in VSMCs, we further studied the involvement of AT_1_R and MasR in the antioxidant actions of spent hen peptides, by the addition of either AT_1_R (losartan) or MasR (A779) antagonists. Results showed that the addition of losartan did not affect the antioxidant effect of any peptide (Figure 6A,B), whereas the presence of A779 partially abolished the antioxidant effect of V-F during a longer period of treatment (24 h) (Figure 7A,B).

### 3.6. Effect of VRP, LKY, VRY, and V-F on Expressions of Antioxidant Enzymes

**Figure 8:**
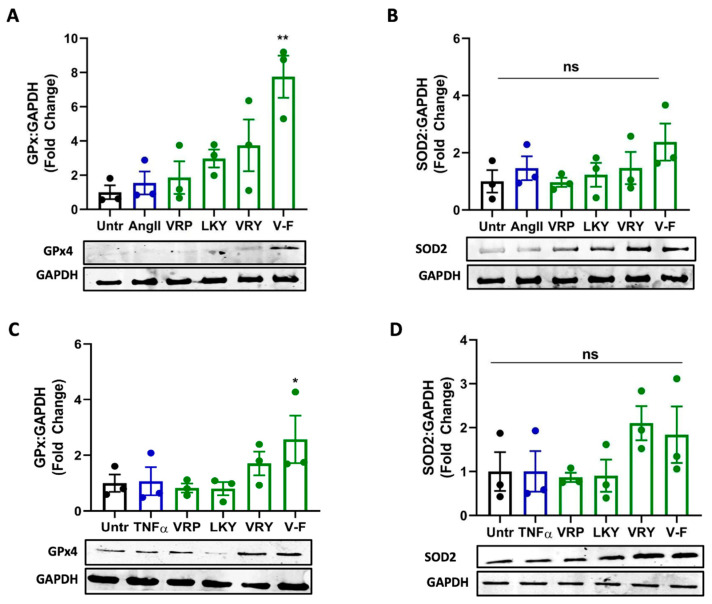
Effect of VRP, LKY, VRY, and V-F on expressions of GPx4 and SOD2. (**A**,**B**) VSMCs were treated with peptides (50 μM) for 24 h before the addition of Ang (1 μM) for 30 min. (**C**,**D**) ECs were treated with peptides (50 μM) for 18 h before the addition of TNFα (10 ng/mL) for 30 min. Protein bands were normalized to GAPDH. Data were expressed as means ± SEM (*n* = 3); ns: not significant (*p* > 0.05); * *p* < 0.05, ** *p* < 0.01, as compared to the Ang II-/TNFα-treated group.

Next, we further studied whether VRP, LKY, VRY, and V-F modulated the expressions of antioxidant enzymes in ECs or VSMCs. As shown in Figure 8, only V-F increased GPx4 levels in both cells (with no significant effect on SOD2 expression). VRP, LKY, and VRY were unable to upregulate GPx4 and SOD2 expression in both vascular cells at the tested concentration in vitro.

## 4. Discussion

ROS, including superoxide, hydrogen peroxide, and hydroxyl radicals, are generated by the incomplete, one-electron reduction of oxygen in biological systems. Overproduction of ROS is the foremost cause of oxidative stress that damages lipid, proteins, RNA, and DNA, and interrupts various cellular functions [10,11,34]. In the vasculature, oxidative stress and associated oxidative damage contribute to endothelial dysfunction and vascular remodeling that induces hypertension [10]. Due to the role of oxidative stress in these pathological responses, treatment with antioxidant agents has been used for hypertension management [17,35]. This study evaluated the antioxidant effect of four ACEi or ACE2u peptides (VRP, LKY, VRY, and V-F) that were previously identified from spent hen muscle proteins, with the aim to explore their multifunctional properties before being administrated to animals for assessment of their in vivo antihypertensive effects. It has been reported that spent hen muscle protein-derived bioactive peptides possess various bioactivities, including immunomodulatory, antihypertensive, anti-inflammatory, and anti-aging properties [27,28,31,36,37]. The revelation of more beneficial health effects of spent hen-derived peptides could enhance value-added uses of spent hens as functional food ingredients. Many antioxidant peptides derived from meat byproducts, such as bone, blood, and skin, have been reported previously [38]; however, among the currently reported antioxidant peptides, a vast majority of them have been evaluated for their antioxidant activities using in vitro, substrate-based biochemical assays, which lack biological relevance [39].

TNFα-stimulated ECs and Ang II-stimulated VSMCs are two commonly-used cellular models to evaluate the cellular antioxidant activity of bioactive peptides [30]. VRP, LKY, VRY, and V-F showed no cytotoxicity in ECs and VSMCs, but exhibited great antioxidant effects in both cells upon stimulation by TNFα or Ang II, respectively (Figure 1, Figure 2 and Figure 3). Both TNFα and Ang II are reported to be substantially elevated in SHRs; therefore, these peptides demonstrated potential in ameliorating hypertension-associated oxidative stress in vivo [10]. Peptides are composed of amino acids, some of which are natural antioxidants as well. As reported, the presence of H and aromatic or cyclic amino acid residues, such as F, Y, and P, as well as N-terminal hydrophobic amino acid residues, such as V and L, contribute to a peptide’s antioxidant activity [40,41]. The imidazole ring of H and the aromatic ring scavenge the radicals by donating a proton, and thus terminate the radical reaction chain. These amino acid residues constitute approximately two-thirds of the peptide chains in this study, and thus might contribute to their ability to quench free radicals directly. The potent antioxidant effects of spent hen peptides further demonstrated that muscle proteins are a rich source of antioxidant peptides [38].

Mechanistically, TNFα induces ROS production through its receptors; Ang II activates ROS production through AT_1_R, whereas elimination of ROS involves MasR that has been characterized to counterbalance harmful effects from the Ang II–AT_1_R axis [42,43]. By evaluating the effect of spent hen peptides on the expression of TNF receptors, we found that none of these peptides altered the expression of TNF-R1/R2 in ECs (Figure 4 and Figure S1). To the best of our knowledge, no studies have reported that peptides can directly block TNF receptors as antagonists in cells or in animals. Therefore, unaltered expression of TNF-R1/R2 indicated that neither of them participate in the antioxidant actions of spent hen peptides in ECs. In VSMCs, however, VRY and V-F downregulated AT_1_R expression, while V-F also upregulated MasR (Figure 5). Previous studies have also reported that peptides like IRW (egg-derived) and LRW (pea-derived) modulate AT_1_R expression or upregulate MasR expression in cells at a similar concentration (50 μM) [22,23], as well as regulate their expression at either the gene or protein level in SHRs, such as IRW, IQP, VEP, LY, RALP, and GHS [44,45,46]; these modulatory effects are thought to be involved in their bioactivities. Since the downregulation of AT_1_R or upregulation of MasR may contribute to reduced oxidative stress, we further studied the role of both receptors in the antioxidant actions of peptides in this study by adding AT_1_R (losartan) or MasR antagonist (A779) prior to Ang II treatment [47,48]. Both peptides were tested for short (1 h) and long (24 h) periods of treatment; the short-time treatment was used to study whether peptides acted as an AT_1_R antagonist or a MasR agonist (since their expression was not altered within 1 h by peptide treatment), whereas the long-time one also considered the altered expressions of AT_1_R and MasR by peptides. Results showed that AT_1_R was not involved in the antioxidant effects of any peptide, but pre-treatment with V-F for 24 h, not 1 h, involved MasR in its antioxidant effect (Figure 6 and Figure 7). This indicated that V-F exerted antioxidant activity that was partially dependent on MasR, and to a larger extent, on the activation of the ACE2–Ang (1–7)–MasR axis, since V-F is an ACE2u peptide [28]. The upregulated ACE2 might degrade Ang II to Ang (1–7), followed by binding with MasR that is also upregulated by V-F, contributing to its antioxidant effect in VSMCs. Upregulation of the ACE2–Ang (1–7)–MasR axis by peptides has recently been reported as an emerging mechanism for mediating their antioxidant and anti-inflammatory activities in cells, as well as for the improvement of vascular function and blood pressure regulation in SHRs [23,32,46]. Given that ACE2 upregulation by bioactive peptides presents a new mechanism mediating the peptides’ beneficial health effects, our results further support the role of ACE2u peptides in ameliorating Ang II-induced pathological responses, which contributes to the treatment of hypertension.

Antioxidant enzymes are a group of proteins involved in transforming ROS into stable and less reactive molecules, thereby protecting cells against oxidative damages [49]. SOD2 is a mitochondrial antioxidant enzyme that terminates superoxide anions, the vital source of redox stress [50]. GPx4 is also an essential mitochondrial antioxidant peroxidase that catalyzes the reduction of hydroperoxides [51]. Increased lipid peroxidation is related to low levels of GPx4, presenting its activation as a mechanism to attenuate oxidative stress and lipid peroxidation [51,52]. Peptides are reported to regress oxidative stress by activating antioxidant enzymes like GPx and SOD. For example, oat-derived peptides exert cytoprotective roles in stressed hepatic HepG2 cells through increasing GPx and SOD levels [53]. Low-molecular-weight, soybean-derived peptides (1000–2000 Da) were reported to suppress the production of ROS and MDA in HepG2 cells stimulated by hydrogen peroxide, which is associated with the upregulated expression of GPx and SOD [19]. In addition, a potato-derived antioxidant dipeptide, IF, significantly increased GPx4 and SOD2 in SHR kidneys and protected the animal against renal damage [20]. Our results demonstrated that only V-F upregulated expression of GPx4 in both VSMCs and ECs (Figure 8A,C). It also increased the SOD2 level in both cells, but the results were statistically insignificant (Figure 8B,D). VRP, LKY, and VRY did not affect the cellular level of GPx4 and SOD2, indicating that their abilities in attenuating superoxide and MDA production were possibly through acting as the direct ROS scavengers. This appeared to align with the result that only MasR was involved in the antioxidant effect of V-F in Ang II-stimulated VSMCs (Figure 7). 

It is imperative to point out the limitations of the present study. For example, V-F reduced Ang II-stimulated oxidative stress in VSMCs, likely by upregulating the ACE2–MasR pathway and elevating antioxidant enzyme levels; however, the interactions between antioxidant enzymes and the ACE2–MasR pathway has not been studied in this work. Furthermore, the involvement of the nuclear factor erythroid 2−related factor 2 pathway on the peptide’s antioxidant activity remains to be elucidated [20,47]. Peptides may also reduce superoxide generation through activation of Ang II type 2 receptor (AT_2_R) in VSMCs; therefore, the role of AT_2_R in the antioxidant effect of spent hen peptides requires exploration [11]. Lastly, except for VRP, the other three peptides, LKY, VRY, and V-F, are susceptible to gastrointestinal degradation, which can significantly influence a peptide’s in vivo efficacy [28]. Thus, despite the cellular antioxidant effects, it remains unknown whether or not these peptides will extend their bioactive function to animals, particularly in the context of hypertension. Future animal study is warranted to address the above-mentioned concerns.

## 5. Conclusions

The antioxidant effects of four previously identified, spent hen-derived peptides, including three ACEi peptides (VRP, LKY, and VRY) and one ACE2u peptide (V-F), were studied in two vascular cells, ECs and VSMCs, in vitro. All the four peptides reduced oxidative stress in both ECs and VSMCs, initiated by TNFα or Ang II stimulation, respectively. None of these peptides altered the expression of TNFα receptors (TNF-R1 and TNF-R2) in ECs, while VRY and V-F downregulated AT_1_R and meanwhile V-F upregulated MasR in VSMCs. In addition, we found that V-F exerted its antioxidant effect partially dependent on MasR in VSMCs, which might involve upregulating ACE2 and counteracting the ROS produced from the binding of Ang II with AT_1_R. Further analysis on the expression of endogenous antioxidant enzymes demonstrated that only V-F upregulated GPx4 and SOD2. In summary, the antioxidant effect of VRP, LKY, and VRY may be through acting as ROS scavengers, whereas the bioactivity of V-F also involves the upregulation of endogenous antioxidant enzymes in ECs and VSMCs.

## Data Availability

The data presented in this study are available upon request.

## Supplementary Materials

The following are available online at https://www.mdpi.com/2076-3921/10/2/290/s1, Figure S1: Effect of VRP, LKY, VRY and V-F (50 μM) for 48 h on expressions of TNF-R1 and TNF-R2.
