# Supplementary material for: Spent Hen Muscle Protein-Derived RAS Regulating Peptides Show Antioxidant Activity in Vascular Cells
Source: Antioxidants (Basel). 2021 Feb 15;10(2):290. doi: 10.3390/antiox10020290 (PMC7919344; doi:10.3390/antiox10020290)
Supplement: Supplementary file 1 [file antioxidants-10-00290-s001.pdf]

Supplementary materials:

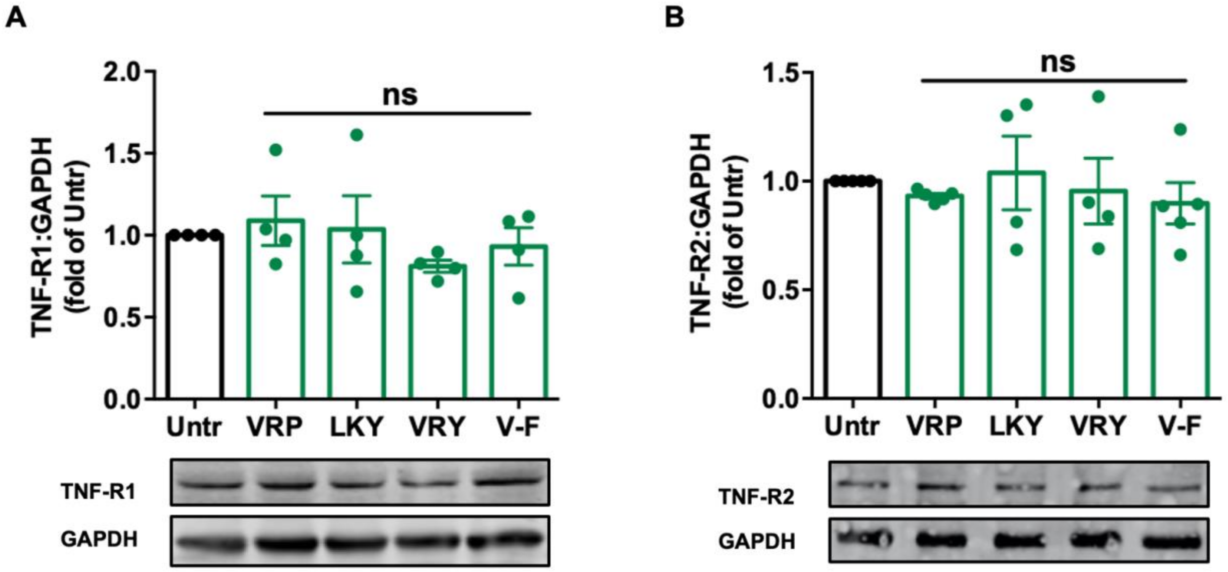

Figure S1. Effect of VRP, LKY, VRY and V-F (50 µM) for 48 h on expressions of TNF-R1 (A) and TNF-R2 (B) in ECs. Protein bands were quantified by densitometry and normalized to GAPDH. Data were expressed as means ± SEMs of 4-5 independent experiments and normalized to the untreated group (Untr). ns, not significant ($p > 0.05$), as compared with the untreated group.
